# Long-term effects of early/late-onset visual deprivation on macular and retinal nerve fibers layer structure: A pilot study

**DOI:** 10.1371/journal.pone.0283423

**Published:** 2023-03-23

**Authors:** Adriano Magli, Paolo Esposito Veneruso, Michele Rinaldi, Roberto Caputo, Fausto Tranfa, Ciro Costagliola

**Affiliations:** 1 Department of Ophthalmology, Orthoptic and Pediatric Ophthalmology, University of Salerno, Salerno, Italy; 2 Division of Ophthalmology, University Hospital “Federico II”, Naples, Italy; 3 Pediatric Ophthalmology Unit, A. Meyer Children’s Hospital, Florence, Italy; 4 Eye Clinic, Multidisciplinary Department of Medical, Surgical and Dental Sciences, University of Campania “Luigi Vanvitelli”, Naples, Italy; 5 Department of Neurosciences, Reproductive and Odontostomatological Sciences, University of Naples Federico II, Naples, Italy; The University of Iowa, UNITED STATES

## Abstract

**Background/aims:**

Tomographic analysis of macular and peripapillary retinal nerve fibers layer (RNFL) thickness in patients with history of congenital (CC) and developmental cataract (DC).

**Methods:**

Analysis of macular and RNFL thickness using a spectral-domain optical coherence tomography was performed. Retinal layers thickness was measured using the internal segmentation software. Measurements of affected (unilateral and bilateral), contralateral eyes and control eyes were compared.

**Results:**

Patients with history of CC or DC (n = 13 and 11 respectively) and 35 healthy control subjects were enrolled. Thicker inner and outer nuclear layers (INL, ONL) and thicker ONL were found when CC and DC group when compared to controls respectively. Bilateral CC showed the most relevant differences. Slight thickening of CC inner retinal layers were found when compared to DC. Increased superonasal RNFL thickness was found in CC group when compared to DC and controls. Thickening of RNFL of contralateral unaffected eyes of unilateral CC were found when compared to controls.

**Conclusion:**

Significant macular and RNFL thickness changes between CC, DC patients and controls that partially involve also contralateral unaffected eyes of unilateral congenital cataract were found. CC and DC groups show significant differences only in inner retinal layers thickness. Our data suggest that early visual deprivation may influence retinal arrangements occurring during development involving predominantly the outer nuclear layer and para/perifoveal inner retinal layers, and confirm that early treatment of CC allow to achieve better long-term visual outcome. Moreover functional and structural data support the hypothesis that unilateral amblyopia is not exclusively an unilateral issue.

## Introduction

The morpho-functional impairment of the lateral geniculate nucleus and visual cortex induced by amblyopia have been widely described [1, 2] and recently supported by functional magnetic resonance imaging studies [3], however, the effects on retinal structures are still a debatable issue. Many studies, based on tomographic evaluation of macula and optic nerve in anisometropic/strabismic patients, reported substantially immature retinal architecture with increased retinal thickness and/or retinal nerve fiber layers (RNFL), reduced pit depth and frequent absence of the bulge of the foveal ellipsoidal zone [4–6]. Szigeti reported an increased outer nuclear layer in ambyopic eyes compared to fellow eyes suggesting the photoreceptors’ involvement [7]. By contrast, no significant differences between amblyopic and contralateral eyes are also described [8–12]. Less common is the analysis of retinal structures in amblyopic patients affected by infantile cataract. To the best of our knowledge, only two studies focused on retinal structure in deprivation amblyopia; Kim et al. [13] reported an increased RNFL in the nasal sector in amblyopic eyes and Wang et al. [14] reported an increased central subfield thickness in patients affected by congenital and developmental cataract with no correlation between this finding and age at surgery or interocular difference for visual acuity. Herein, we report preliminary data on the tomographic assessment of macular and peripapillary RNFL thickness in patients with history of congenital and developmental cataract.

## Materials and methods

Twenty-four patients with history of congenital (CC—defined as a lens opacity present at birth or within 6 month of age) and developmental cataract (DC—defined as a lens opacity developed later than 6 month of age) were selected from a larger cohort of approximately 150 patients. All patients had undergone an uncomplicated cataract surgery procedure with intraocular lens (IOL) implant (primary IOL implant in all DC patients, secondary IOL implant in all CC patients at 2,5 years of age, approximately) before inclusion in the study. Briefly, surgical procedures consisted of mechanical anterior capsulorhexis, automated extracapsular lens extraction, posterior capsulorhexis or posterior capsulotomy and central anterior vitrectomy performed under general anaesthesia [15, 16].

Thirty-five healthy age-matched patients with refractive error (spherical equivalent) within ±2.5D and BCVA 0.0 LogMAR or better in both eyes, were enrolled and assigned to control groups (C).

Table 1 shows demographic and clinical data including age at surgery, age at examination, best corrected visual acuity (BCVA), ocular motility disorders and morphology of cataract.

**Table 1:** Demographic and clinical data.

	n (eyes)	Age at examination (years)	age at surgery (years)	Axial length (mm)	BCVA (logMAR)		cataract morphology	motilty disorders
**C**	35	13.9±5.5	-	23.6±0.8	-0.18		-	-
**CC**	16	13.2±3.9	0.8±0.6	23.3±2	0.47±0.4		6 total, 3 nuclear + posterior polar, 4 lamellar	5 congenital exotropia, 3 exotropia + hypertropia, 4 congenital esotopia
**bCC**	6	15.9±0.7	0.3±0.1	22.2±1.1	0.16±0.1			
**uCC**	10	12.4±4.2	1.1±0.6	23.9±2.1	0.65±0.4	-0.10±0.1		
**DC**	17	13.6±5.9	6.5±4.0	23.4±1.4	0.00±0.1		3 anterior polar, 4 zonular, 4 lamellar	1 esophoria, 3 alternating esotropia, 2 intermittent exotropia, 1 infantile exotropia
**bDC**	12	16.14±6.1	7.3±4.5	23.1±0.9	-0.03±0.1			
**uDC**	5	11.5±3.2	5.2±2.9	23.8±2	0.06±0.2	-0.12±0.1		

mean±standard deviation

C—controls; CC—congenital cataract; bCC—bilateral CC; uCC—unilateral CC; DC—developmental cataract; bDC—bilateral DC; uDC—unilateral DC.

All patients underwent extensive ophthalmologic and orthoptic characterization, including BCVA, measured with the Early Treatment Diabetic Retinopathy Study (ETDRS) charts, expressed as a logarithm of the minimum angle of resolution (log-MAR), slit-lamp biomicroscopy, intraocular pressure (IOP) measurement, indirect ophthalmoscopy, retinoscopy, autorefractometry, Hirschberg test, cover and cover-uncover test, Lang’s stereotest, Worth’s 4 light test, axial length (IOLMaster 500 –Carl Zeiss AG, Oberkochen, Germany) and retinal spectral-domain (SD) optical coherence tomography (OCT) (Spectralis SD OCT, Heidelberg Engineering, Inc., Heidelberg, Germany). Patients’ selection was performed on the basis of the following exclusion criteria: presence of retinal (i.e. oedema, dystrophy, atrophy, retinopathy of prematurity), corneal or optical media disease (i.e. opacities, oedema), ocular abnormalities (i.e. microphthalmos, posterior- type persistent hyperplastic primary vitreous, persistent fetal vasculature), history of congenital glaucoma or systemic disorders able to influence learning ability, postoperative complications (i.e. retinal detachment, macular oedema, ocular inflammation), glaucoma and presence of nystagmus or fixation instability.

**Fig 1:**
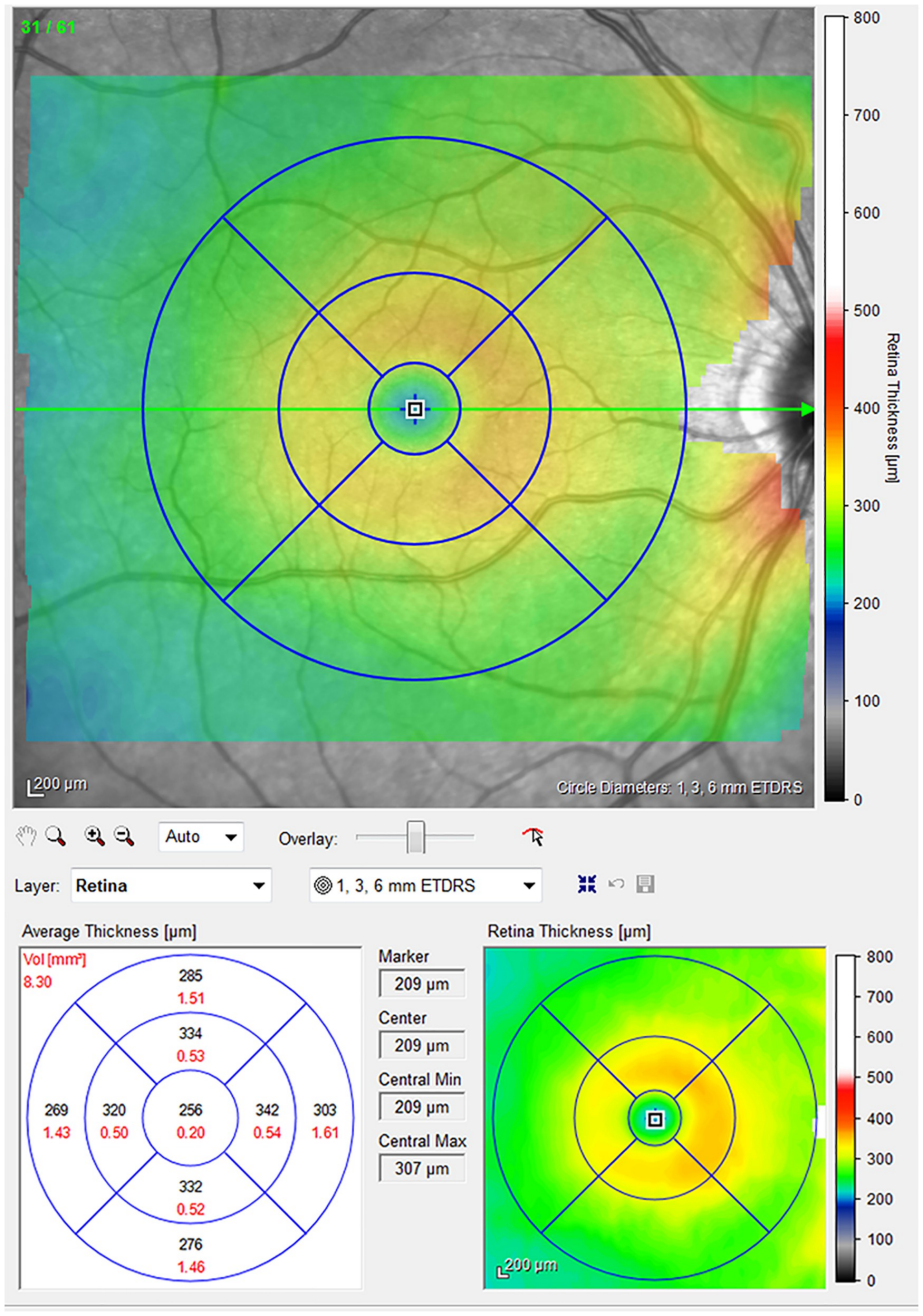
Retinal thickness analysis algorithm in which mean thicknesses (in micrometers) are provided for each of 9 areas defined by an Early Treatment Diabetic Retinopathy Study (ETDRS).

**Fig 2:**
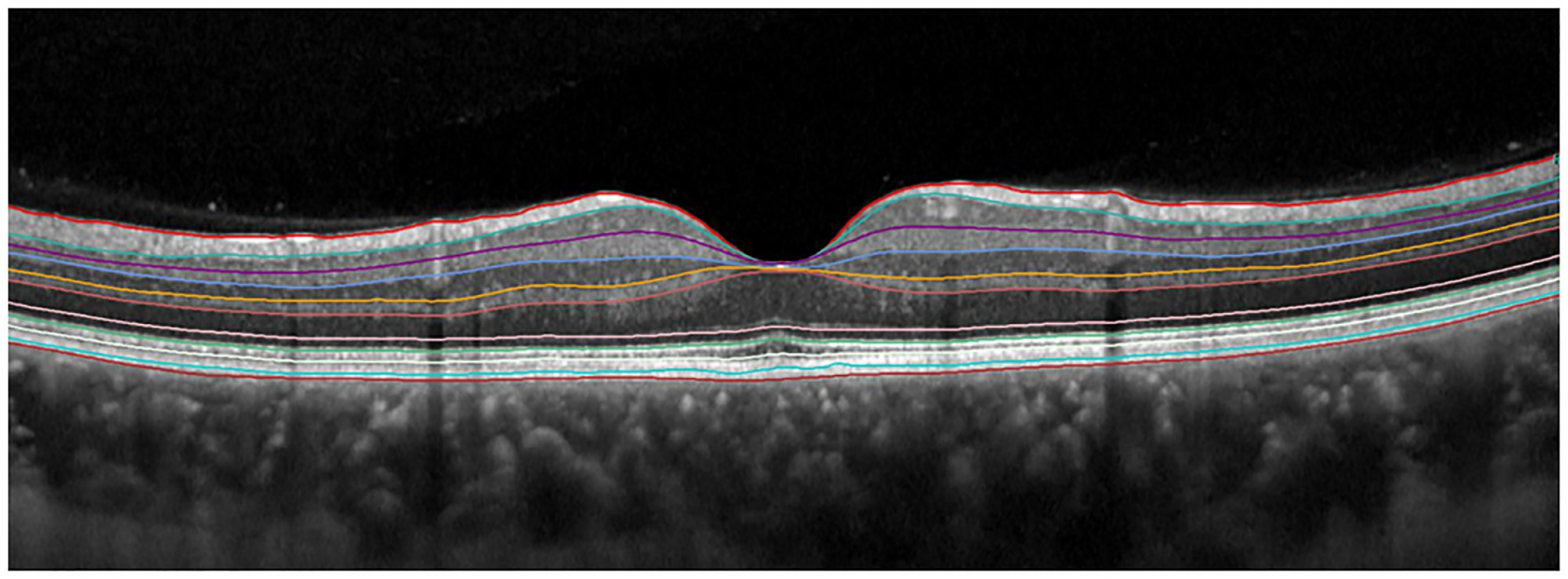
Representative automatic retinal layers segmentation.

Macular thickness analysis was performed in all patients using the internal fixation target, a 30x25° grid with 61 horizontal axial scans. Images with a quality index <25 were excluded. Retinal thickness analysis algorithm provided mean thickness analysis of 9 areas, defined by the Early Treatment Diabetic Retinopathy Study (ETDRS) circle for every layer (Fig 1). The internal automated segmentation software (Fig 2) was used to obtain individual retinal layer thickness measurements including: overall retinal thickness (RT), nerve fibers layer (NFL), ganglion cell layer (GCL), inner plexiform layer (IPL), inner nuclear layer (INL), outer plexiform layer (OPL), outer nuclear layer (ONL), and retinal pigment epithelium (RPE). Total retinal and single retinal layer thickness were analyzed at central 1mm, 3 and 6 mm of eccentricity in each sector (*n*- nasal, *i*- inferior, *t*- temporal and *s*- superior).

RNFL thickness analysis was performed with 3.5, 4.1 and 4.7 mm circle scan and thickness of each sector (*g-* global, *in-* inferonasal, *it-* inferotemporal, *st-* superotemporal and *sn-* superonasal) was analyzed.

Comparisons between macular and RNFL thickenss of congenital cataract, development cataract, contralateral eyes in unilateral CC and DC group (CCc and DCc respectively), controls, bilateral and unilateral CC and DC were performed. Only right eyes of control group were considered for statistical analysis.

The study followed the tenets of the Declaration of Helsinki and an informed consent to participate in the study was obtained from all subjects, or their parents, after full explanation of the aims and modalities of the investigation. This study received an approval from Institutional Review Board of Molise University (n° 19/2021).

### Statistical analysis

Data were tested for normal distribution by Kolmogorov-Smirnov test and are expressed as mean ± one standard deviation. Comparison among groups were performed by one-way analysis of variance with post-hoc analysis (Bonferroni’s correction). The difference between groups were assessed by Student’s t-test. Pearson’s correlation was applied to examine a possible association among BCVA data, time at surgery and retinal/RNFL thickness. The dimension of the study population comprised of 33 eyes was considered adequate to fulfill the needs of this pilot study. For all analyses a p-value <0.05 was considered significant. A commercial statistical software was used (MedCalc^®^).

## Results

Macular (total and each single layer) and retinal nerve fiber layer (RNFL) thickness analysis was performed in all patients. Thirteen patients (16 eyes) were affected by congenital cataract (CC group), 3 patients affected by bilateral CC (6 eyes—bCC group), and 10 by unilateral CC (10 eyes—uCC); 11 patients (17 eyes) were affected by development cataract (DC group), 6 patients affected by bilateral DC (12 eyes—bDC), and 5 by unilateral DC (5 eyes—uDC); 35 healthy, age matched patients were enrolled as controls (35 eyes). BCVA mean values were significantly worse in CC and DC group (CC<DC<C; p<0.001), in bCC and uDC (p<0.001) and in CCc (p = 0.025) when compared to controls. Unilateral congenital cataracts showed the lowest BCVA when compared to all groups (p<0.001). Significant differences between affected and contralateral eyes were found exclusively in CC group (p<0.001).

No statistically significant differences were found in axial length mean values between groups (p = 0.256). Clinical and demographic data are shown in Table 1.

Retinal thickness analysis showed significant differences between CC, CCc, DC and DCc groups involving: ONL within central 1 mm, total retina (*all sectors*), NFL (*n-*), IPL (*n-*, *i-*), INL (*n-*, *s-*), ONL (*i-*, *t-*, *s-*) within central 3 mm and total retina (*n-*, *s-*), NFL (*i-*), INL (*n-*, *s-*), ONL (*i-*, *t-*, *s-*) within central 6 mm. Significant retinal thickness differences were found between uCC, bCC, uDC and bDC groups, involving: OPL and ONL within central 1 mm, total retina (*n-*, *i-*, *s-*), IPL (*n-*), INL (*n-*), ONL (*all sectors*) within central 3 mm and total retina (*n-*, *i-*, *s-*), INL (*n-*), ONL (*all sectors*) within central 6 mm.

Significant differences of RNFL thickness between CC, CCc, DC and DCc groups involved the *sn-* sector at 3.5, 4.1 and 4.7 mm, whereas, between uCC, bCC, uDC and bDC groups involved the *sn-* sector at 3.5mm and the *s-* and s*n-* sectors at 4.7 mm.

**Fig 3:**
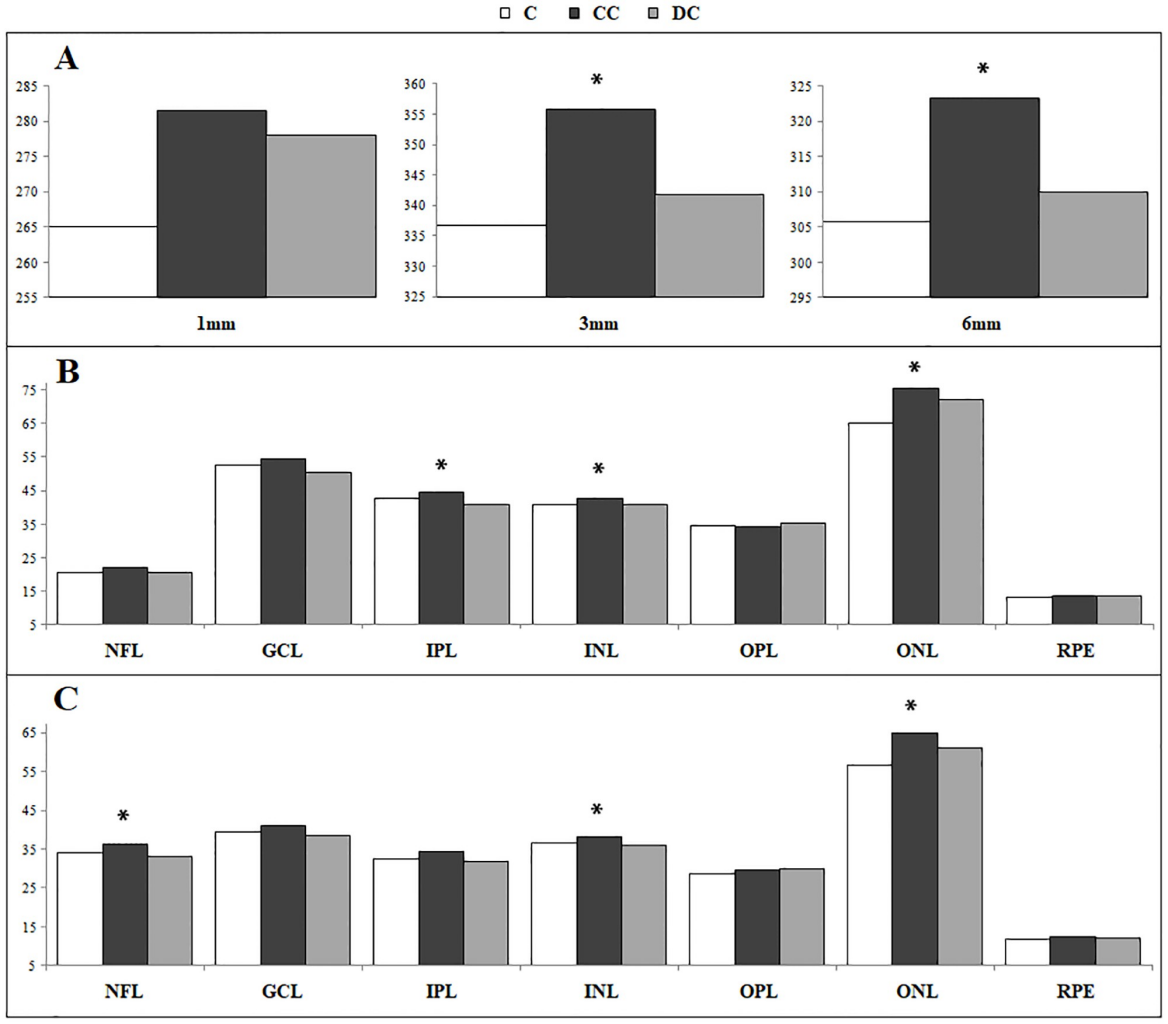
Representative distribution of total retinal thickness (micron). Retinal thickness in control (C), congenital cataract (CC) and development cataract (DC) groups whithin 1, 3 and 6 mm of eccentricity (A); thickness of nerve fibers layer [NFL], ganglion cells layer [GCL], inner plexiform layer [IPL], inner nuclear layer [INL]. outer plexiform layer [OPL], outer nuclear layer [ONL], retinal pigment epithelium [RPE], at 3 (B) and 6 mm (C) of eccentricity. * statistically significant.

**Fig 4:**
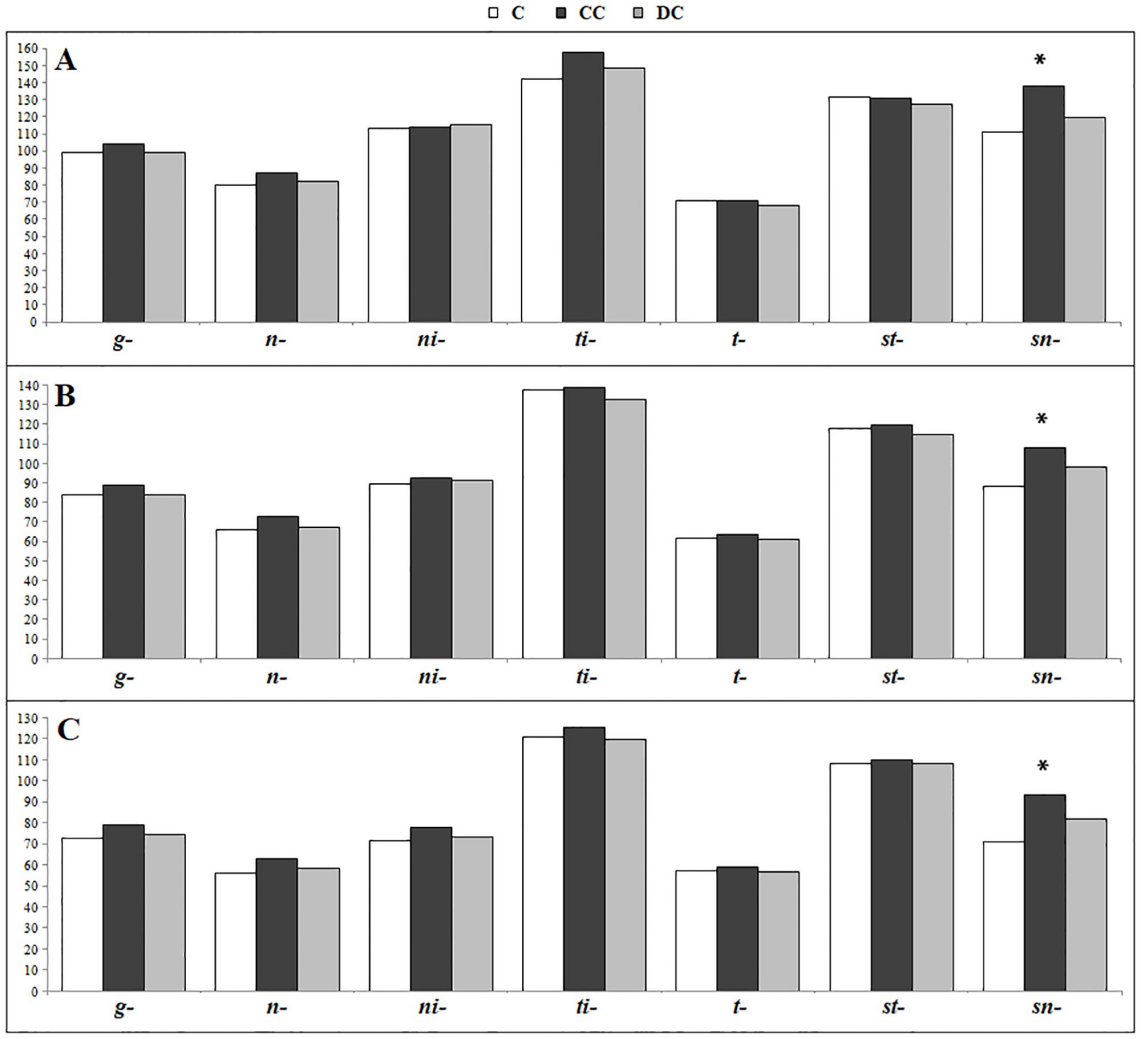
Representative distribution of total RNFL thickness (micron). RNFL thickness in control (C), congenital cataract (CC) and development cataract (DC) groups at 3.5 (A), 4.5 (B) and 4.7 mm (C) of eccentricity; thickness of *g-* global, *in-* inferonasal, *it-* inferotemporal, *st-* superotemporal and *sn-* superonasal sectors are shown. * statistically significant.

Schematic representation of retinal and RNFL thickness mean values are shown in Figs 3 and 4.

CC group showed strong correlation between age at surgery and BCVA (p<0.0001). CC group showed a inverse global trend of correlation between age at surgery, para/perifoveal layers thickness and BCVA and slight correlation between RNFL thickness, age at surgery and BCVA. DC group showed significant inverse global trend of correlation between age at surgery and retinal layers thickness, between RNFL thickness and age at surgery and between central macular thickness and BCVA. Complete data of retinal and RNFL thickness, comparison and statistically significant correlations are shown in supplemental material.

## Discussion

The role of retina in the visual pathways impairment occurring in patients affected by amblyopia, still represent a controversial issue. Retinal structure in anisometropic or strabismic amblyopia (or both) has been widely described using SD-OCT. Although some authors described slight, no significant differences between amblyopic and fellow eyes [8–12, 17], the most common finding in amblyopic patients is an increase of the central macular/foveal thickness [4–6, 18–20].

Moreover, morphological differences in amblyopic eyes have been described when compared to control ones: increased foveal thickness, reduced pit depth (measured along the horizontal meridian), flattening of nasal and temporal side of foveal pit [4] and photoreceptor outer segment elongation [21]. Studies describing macular structure by using segmentation software reported marginal peripheral alterations of some retinal layer (nasal NFL and inferior INL) with no identifiable pattern [22] and increased central ONL thickness in amblyopic eyes compared to controls [7]. No significant abnormalities are commonly described in RNFL thickness [4, 9, 10, 23, 24]. By contrast, some studies reported an increased circumpapillary RNFL thickness in amblyopic when compared to fellow eyes [6, 20].

### Morphological findings

Only two studies focused on macular and RNFL structure in deprivation amblyopia, taking into account patients affected by pediatric cataract. Kim et al. [13] studied 14 pseudophakic patients comparing macular and RNFL thickness of amblyopic, fellow and control eyes. No differences were found in central macular thickness among groups. Amblyopic eyes tend to have an increased thickness in inner-temporal, outer-superior and outer-inferior macular sectors, when compared to the fellow, non-amblyopic eyes. Peripapillary RNFL thickness of amblyopic eyes was significantly thicker in the nasal sector compared to fellow non-amblyopic and healthy control eyes. Wang et al. [14], analyzing 40 pseudophakic patients (21 unilateral, 19 bilateral) affected by congenital and developmental cataract, described an increasing of macular central subfield thickness in amblyopic eyes; no correlation with age at surgery or interocular difference for visual acuity was found.

In this study comparison of macular thickness in congenital cataract and controls showed an increased thickness of inner and outer nuclear layers and superonasal sector of peripapillary RNFL, according to Wang’s findings. Developmental cataract showed increased ONL thickness compared to controls, whereas no significant difference was found in RNFL thickness. Congenital cataract showed a slight significant thickening of para/perifoveal inner retinal layers (NFL, IPL and INL) compared to developmental ones whereas, no significant differences were found in RNFL thickness. The analysis of unilateral and bilateral cataract showed that retinal thickness differences were more pronounced in bCC and ONL thickening was found, mostly, in both bCC and bDC. By contrast, uCC showed thicker RNFL in nasal and superonasal sectors compared when compared to all groups. Thickening of ONL was previously reported by Szigeti [7] in patients with anisometropic/strabismic amblyopia. Both CC and DC showed slight differences of inner retinal layers thickness suggesting a different effects on the development of retinal structures induced by onset and removal timing of visual deprivation.

Coherently with abnormal functional findings of the fellow eyes in unilateral amblyopia reported by Birch and other Authors [24–26], we found slight differences when RNFL thickness of CCc were compared to controls, suggesting the occurrence of micro-structural changes in fellow eyes.

Previous histological and tomographic studies focused on foveal development from mid-gestation to maturity, showed how cones packing and elongation, increasing cones density, thickening of central photoreceptors layer, inner retinal cells migration from fovea to mid-periphery and presumably retinal stretch during eye growth, lead to pit shape widening and shallowing up to complete maturation (approximately 13 years of age) [27–30]. The onset of an early visual deprivation during development would let think to an interruption of a physiological maturation stage such as ONL thickening, but the increase of ONL thickness in subjects with history of pediatric cataract, allows us to speculate that the macular maturation process in early visually-deprived patients might be normally triggered but inappropriately continue during time as if inhibitory inputs were, at least partially, defective.

Post-natal retinal modifications involved parafoveal and mid-peripheral retina as well, including thickening of GCL and INL due to peripheral-ward migration of GC from foveal slope, and earlier maturation of parafoveal photoreceptors compared to foveal ones [28].

Consistently with these findings, we found significant correlation between age at surgery and retinal thickness, involving meanly para/perifoveal inner layers, whose development appear to be much more dynamic than foveal one [30], in both CC and DC group and slight relationship between age at surgery and RNFL thickness (more evident in DC group).

### Functional findings

Our results showed BCVA of CC and DC were both worse than controls (CC<DC), only CC group showed significant differences between affected and contralateral eyes. Moreover contralateral unaffected eyes BCVA in congenital cataract were significantly worse than control as well. This finding is consistent with previously mentioned studies describing functional abnormalities of fellow eye in unilateral amblyopia.

Functional data described by Lewis and Maurer [31] in early visually-deprived children affected by dense cataract occurred at different age, allowed to identify multiple sensitive period in visual development, in particular, the period of visually-driven normal development, the sensitive period for damage, and the sensitive period for recovery. According to their results and previously literature [32] we found a strong correlation (p<0.0001) between age at surgery and BCVA in CC group, supporting that early visual deprivation removal lead to a better log-term functional outcome. Moreover, the slight impairment of BCVA found in DC group compared to controls support the hypothesis of a visual acuity sensitive period for damage lasting up to 10 years of age [31]. Unexpectedly, bCC showed the most relevant retinal differences when compared to all groups (C, uCC, uDC and bDC) and ONL thickening was found, predominantly, in bilateral forms of cataract. The most relevant RNFL thickening was found in uCC. Considering functional data and the lack of strong correlation between BCVA and RNFL, these findings would suggest that visual deprivation may be the cause of retinal thickness modifications independently of amblyopia depth. Correlation between BCVA and retinal thickness appeared to have different pattern between CC and DC group, related to para/perifoveal zone and central fovea respectively. These relationship might relate the role of mid-peripheral retina and foveal refinement during first years of life and the development of BCVA in congenital and developmental cataract respectively.

Interestingly, previous electrophysiological studies in patients affected by congenital and developmental cataracts showed significant impairment of cone-driven retinal function presumably triggered from photoreceptors dysfunction and involving inner retinal layers [33]. These findings were more evident in congenital cataract than developmental ones when compared to controls, and were consistent with structural differences found in this paper involving the ONL more than inner retinal layers [34].

This study presents several limitations including (a) limited sample size that (b) make impossible the comparisons of subgroups based on depth of amblyopia, age or visual deprivation persistence time, (c) lack of data regarding cataract etiology (d) lack of cataract density scale, (e) the role of strabismus on depth of amblyopia and retinal development might have influenced retinal/RNFL thickness measurements (f) the possible role of variability of retinal layers thickness measurement related to accuracy of segmentation cannot be completely excluded. Nevertheless it represent one of the very few tomographic studies focused on retinal morphology in patients with history of congenital and developmental cataract. Our results show significant macular and RNFL thickness changes between patients with history of congenital and developmental cataract and controls that partially involve also contralateral unaffected eyes of unilateral congenital cataract. Congenital and developmental cataract groups show significant differences only in inner retinal layers thickness. Our data suggest that early visual deprivation may influence retinal arrangements occurring during development involving predominantly the outer nuclear layer and para/perifoveal inner retinal layers, and confirm that early treatment of congenital cataract allow to achieve better long-term visual outcome. Moreover, functional and structural data support the hypothesis that unilateral amblyopia is not exclusively an unilateral issue.

## Supporting information

S1 File Complete macular thickness mean values at 1, 3 and 6 mm. (DOCX)

S2 File Complete RNFL thickness mean values at 3.5, 4.1 and 4.7mm. (DOCX)

S3 File Significant correlations between Retinal layers thickness, RNFL thickness, BCVA and age at surgery. (DOCX)
