# Supplementary material for: Long-term effects of early/late-onset visual deprivation on macular and retinal nerve fibers layer structure: A pilot study
Source: PLoS One. 2023 Mar 23;18(3):e0283423. doi: 10.1371/journal.pone.0283423 (PMC10035877; doi:10.1371/journal.pone.0283423)
Supplement: S1 File — (DOCX) [file pone.0283423.s001.docx]

| **S1 –** Complete macular thickness mean values at 1, 3 and 6 mm. | | | | | | | | | | | | |
| --- | --- | --- | --- | --- | --- | --- | --- | --- | --- | --- | --- | --- |
|  |  | **C** | **CC** | **CCc** | **DC** | **DCc** |  | **uCC** | **bCC** | **uDC** | **bDC** |  |
|  | | | | | | | | | | | | |
| **1mm** | | | | | | | | | | | | |
| **TOT** | | 265.1±24.2 | 281.5±23.3 | 268.7±16.6 | 278±24.7 | 265.7±19.8 |  | 284.4±20.7 | 276.7±28.6 | 282.2±27.8 | 275.5±23.9 |  |
| **NFL** | | 11.7±2.1 | 13±2.4 | 12.2±1.4 | 12.6±2 | 12±1.4 |  | 13.4±2.1 | 12.3±3 | 12.5±2.3 | 12.6±1.9 |  |
| **GCL** | | 14.8±3.9 | 18.4±6.4 | 16.8±4.9 | 16.4±4.4 | 15.2±4.5 |  | 20.1±6.7 | 15.7±5.2 | 18.3±4.8 | 15.3±3.9 |  |
| **IPL** | | 20.6±4.8 | 21.7±5.1 | 21.2±4.1 | 21.3±3.4 | 20.7±2.7 |  | 22.7±4.6 | 20±5.8 | 22.3±3.8 | 20.6±3.1 |  |
| **INL** | | 16.6±4.6 | 20.3±6.7 | 18.6±4.2 | 19.4±4.5 | 16.7±2.2 |  | 22.7±6.5 | 16.3±5.5 | 20±1.8 | 19±5.6 |  |
| **OPL** | | 25.7±6.1 | 24.4±6.8 | 24.4±4.7 | 25.1±7.3 | 27.7±7.9 |  | 26.2±6.6 | 21.5±6.5 | 31±8.1 | 21.5±3.8 | * |
| **ONL** | | 87.1±5.7 | 96.4±7.2 | 86.3±6.3 | 95.8±10.5 | 85±4.9 | * | 94.6±8 | 99.3±4.8 | 90.5±14 | 99±6.7 | * |
| **RPE** | | 16.1±2.2 | 15±1.7 | 16.2±1.1 | 15.5±1.8 | 16.3±2.5 |  | 15.1±1.7 | 14.8±1.8 | 15.8±1.7 | 15.3±1.9 |  |
|  | | | | | | | | | | | | |
| **3mm** | | | | | | | | | | | | |
| **TOT** | *n-* | 340.7±15.4 | 363.1±11.9 | 345.1±13.4 | 346.2±14.6 | 336.3±9 | * | 358.1±6.4 | 371.5±14.7 | 347.8±12.7 | 345.2±16.2 | * |
|  | *i-* | 336.5±16.2 | 355.3±14.9 | 342.5±8.5 | 339.8±17 | 335.2±9.9 | * | 347.7±11.3 | 368±11.4 | 340.2±16.6 | 339.5±18.1 | * |
|  | *t-* | 327.4±15.5 | 343.4±14.1 | 331.1±10.4 | 333.2±15.4 | 323.7±7.2 | * | 341.1±13 | 347.3±16.3 | 332.7±12.9 | 333.5±17.4 |  |
|  | *s-* | 342.6±14.9 | 361.2±15.1 | 344.8±11.4 | 347.7±15.9 | 336.8±6.4 | * | 355.7±10.2 | 370.3±18.4 | 350±9.2 | 346.3±19.2 | * |
| **NFL** | *n-* | 20.1±1.5 | 21.3±2.8 | 19±1.8 | 19.9±1.6 | 18.5±1.5 | * | 21.9±3.3 | 20.3±1.6 | 20.5±1 | 19.4±1.8 |  |
|  | *i-* | 23±2.1 | 25.1±3.5 | 23.3±2.2 | 23.4±3.3 | 22.3±2.5 |  | 25±4 | 25.3±2.7 | 23.8±2.5 | 23.2±3.8 |  |
|  | *t-* | 16.2±1.3 | 16.8±1.9 | 16±0.7 | 16.4±1.3 | 15.5±1.4 |  | 17.3±2.2 | 16±0.9 | 16.3±1.5 | 16.5±1.3 |  |
|  | *s-* | 23.6±1.7 | 25.2±5 | 22.7±3 | 23.3±3.9 | 21.5±2.3 |  | 25.6±6.2 | 24.5±2.4 | 25.3±4.5 | 22.1±3.1 |  |
| **GCL** | *n-* | 52.6±3.5 | 55.3±4.2 | 53.5±7 | 50.1±8.3 | 49.3±4.2 |  | 55.4±4.2 | 55.2±4.6 | 53±4.4 | 48.1±9.9 |  |
|  | *i-* | 53.5±5.1 | 54.6±5 | 54.6±4.2 | 49.8±7.8 | 52.7±5.4 |  | 53.8±5.8 | 56±3.2 | 51.3±5.3 | 48.9±9.2 |  |
|  | *t-* | 49.5±3.6 | 51.9±4.8 | 51.3±5.7 | 48.7±7.1 | 47.2±4.3 |  | 52.7±3.6 | 50.5±6.6 | 49.7±3.5 | 48.1±8.7 |  |
|  | *s-* | 55.1±4.2 | 55.8±5.7 | 55.2±5.4 | 52.4±8.2 | 51.3±5 |  | 55.3±6.8 | 56.5±3.7 | 54.2±5.4 | 51.3±9.6 |  |
| **IPL** | *n-* | 43.4±2.6 | 45.4±3 | 43.9±4.4 | 41.1±4.9 | 41.3±1.4 | * | 45.7±3.2 | 45±2.9 | 42.8±2 | 40±6 | * |
|  | *i-* | 41.9±2.8 | 44.7±3.8 | 43.5±3.1 | 40.2±5.3 | 36.2±11.6 | * | 44.2±4.2 | 45.5±3.3 | 41.3±2.2 | 39.5±6.6 |  |
|  | *t-* | 42.4±2.8 | 43.7±4.3 | 42.8±3.6 | 40.9±4.9 | 40.8±2.6 |  | 44.5±4.4 | 42.3±4.3 | 41.5±2.4 | 40.6±6 |  |
|  | *s-* | 42.9±2.6 | 44.2±4.2 | 43.4±3.7 | 41.3±4.8 | 40.5±2.7 |  | 43.5±4.4 | 45.3±3.8 | 42±2.8 | 40.8±5.8 |  |
| **INL** | *n-* | 40.1±4.3 | 45.5±4.2 | 42.1±5.7 | 42.1±3 | 38.2±1.8 | * | 45.7±3.3 | 45.2±5.8 | 41.7±3.1 | 42.3±3 | * |
|  | *i-* | 42.6±4.7 | 41.8±8.8 | 42.1±2.7 | 41.9±3.3 | 40.3±2.4 |  | 39.4±10.2 | 45.8±3.3 | 40.5±3.2 | 42.7±3.3 |  |
|  | *t-* | 39.1±3.3 | 40.1±3.8 | 40.7±3.6 | 38.8±2.7 | 38.2±2.4 |  | 40.1±3.7 | 40±4.5 | 37.3±2.1 | 39.7±2.7 |  |
|  | *s-* | 41.6±2.8 | 43.9±3.3 | 43.1±3 | 41.1±2.3 | 41.3±1.6 | * | 43.8±2.7 | 44±4.3 | 41.2±1.7 | 41.1±2.7 |  |
| **OPL** | *n-* | 33.8±5.1 | 39.2±14.3 | 33±9.1 | 39±11.2 | 37.2±11.8 |  | 40.9±13.1 | 36.3±17 | 47.5±13.8 | 33.3±3 |  |
|  | *i-* | 40.3±12 | 35.1±6 | 31.9±3.8 | 39.6±10.4 | 38.2±10.5 |  | 36.1±7 | 33.5±3.6 | 44.7±12.5 | 36.6±8.1 |  |
|  | *t-* | 32.4±5.5 | 29.9±2.7 | 32.1±4.7 | 31.9±5.5 | 34.5±7.1 |  | 28.3±1.2 | 32.7±2.3 | 32±4.5 | 31.9±6.2 |  |
|  | *s-* | 31.7±6 | 32.1±7.6 | 34.7±5.9 | 30.2±2.6 | 36.2±6.9 |  | 30.6±2.9 | 34.5±12.1 | 30.3±3.1 | 30.1±2.5 |  |
| **ONL** | *n-* | 66.9±8.1 | 73.1±20 | 71.3±14 | 71.3±16.7 | 69±16.7 |  | 67.3±20.9 | 82.8±15.3 | 59.5±21.3 | 79.2±5.8 | * |
|  | *i-* | 56.4±11.4 | 71.3±12.2 | 68±7.9 | 64.4±14.3 | 60±17.9 | * | 67±13.7 | 78.5±3.4 | 57.5±19.6 | 68.5±8.8 | * |
|  | *t-* | 68.2±7.2 | 79.6±6.3 | 68.2±7.6 | 75±8.4 | 65.3±5.5 | * | 78.9±7.4 | 80.7±4.2 | 74.3±7.7 | 75.4±9.1 | * |
|  | *s-* | 68.6±9.1 | 77.4±10.2 | 67.1±7.7 | 77.9±5.3 | 65±6.5 | * | 75.1±10.6 | 81.2±8.9 | 75.8±4.5 | 79.1±5.6 | * |
| **RPE** | *n-* | 13.7±1.8 | 13.8±1.4 | 14.4±1.1 | 14.4±1.7 | 14.5±0.8 |  | 13.3±0.9 | 14.7±1.6 | 15±1.7 | 14±1.7 |  |
|  | *i-* | 13±1.2 | 13.4±1.9 | 13±0.7 | 13.5±1.2 | 13.3±1 |  | 12.7±1.6 | 14.5±1.9 | 13.8±1.7 | 13.3±0.8 |  |
|  | *t-* | 13±1.6 | 13.1±1.6 | 12.3±0.8 | 12.8±1.2 | 13±0.6 |  | 12.4±1.1 | 14.3±1.6 | 13.2±1.5 | 12.6±1.1 |  |
|  | *s-* | 13.4±1.5 | 13.6±2 | 13.1±0.7 | 13.8±1.6 | 15.5±4.2 |  | 12.9±1.4 | 14.8±2.5 | 14.7±1.6 | 13.3±1.3 |  |
|  | | | | | | | | | | | | |
| **6mm** | | | | | | | | | | | | |
| **TOT** | *n-* | 320±12.9 | 341.4±15.7 | 326.3±5.8 | 325.4±21.7 | 324.7±11.4 | * | 332.7±11.2 | 355.8±10.8 | 329.3±14.1 | 323.1±25.7 | * |
|  | *i-* | 301.2±12.2 | 319.5±18.6 | 312.1±8.4 | 308.9±21.5 | 307.5±9.8 |  | 311.5±18 | 332.8±10.5 | 309±17 | 308.8±24.7 | * |
|  | *t-* | 290.6±32.4 | 302.3±11.3 | 289.1±9.8 | 290.9±13.2 | 287.7±7.8 |  | 296.9±10.4 | 311.2±6 | 294.7±12 | 288.7±13.9 |  |
|  | *s-* | 311.4±13.6 | 329.8±16.7 | 311.4±6.5 | 314.8±18.5 | 310.5±16.2 | * | 326.3±17.3 | 335.7±15.1 | 317.7±14 | 313±21.3 | * |
| **NFL** | *n-* | 46.9±4 | 50±8.4 | 45.8±7.5 | 44.6±8.9 | 43.8±5.3 |  | 51.1±9.7 | 48.2±6.1 | 50.5±4.6 | 41.1±9.1 | * |
|  | *i-* | 35.8±3.4 | 38.3±4.7 | 36.4±3.4 | 33.9±5.6 | 32.7±3.5 | * | 37.7±5.9 | 39.3±1.6 | 36.7±2 | 32.2±6.4 | * |
|  | *t-* | 17.5±1.2 | 19.1±2.4 | 17.8±0.9 | 18.4±1.6 | 17.3±1.2 |  | 19.6±2.9 | 18.2±1 | 19±2.1 | 18.1±1.3 |  |
|  | *s-* | 36.3±5.3 | 38±8 | 32.4±5.1 | 35.4±6.6 | 32.2±5.2 |  | 38.5±9.7 | 37.2±4.6 | 39±6.6 | 33.2±5.8 |  |
| **GCL** | *n-* | 40.7±3.3 | 42.1±4.6 | 41.8±1.9 | 39.8±6.7 | 41.7±4.8 |  | 40.4±4.6 | 45±2.8 | 39.7±3.1 | 39.8±8.4 |  |
|  | *i-* | 39.6±3.5 | 40.4±4.7 | 42.1±3 | 38.9±7.3 | 41.5±2.3 |  | 39.4±5.5 | 42.2±2.5 | 39±4.6 | 38.8±8.8 |  |
|  | *t-* | 37.6±4.1 | 39.8±3 | 39.5±4.2 | 37.1±5.9 | 36±5.5 |  | 39±3.3 | 41.2±2 | 39.2±4.6 | 35.9±6.5 |  |
|  | *s-* | 40.2±2.9 | 41.3±4.9 | 40.8±2.2 | 38.3±5.9 | 40.2±4.7 |  | 41.5±5.7 | 40.8±3.8 | 38.5±4.9 | 38.2±6.7 |  |
| **IPL** | *n-* | 31.9±2.6 | 34.6±3.4 | 33.1±1.7 | 31.4±5.1 | 32.7±3.4 |  | 33.1±2.5 | 37±3.6 | 31.8±2.5 | 31.2±6.3 |  |
|  | *i-* | 31.8±2.7 | 34.2±3.4 | 33.9±2.4 | 32±5.6 | 34±3 |  | 33.5±3.1 | 35.3±3.7 | 32.3±3.3 | 31.8±6.8 |  |
|  | *t-* | 33.2±2.9 | 34.7±1.9 | 33.6±2.6 | 32.7±3.2 | 33±2.1 |  | 34.1±1.5 | 35.7±2.3 | 34.2±2.3 | 31.8±3.5 |  |
|  | *s-* | 32.6±2.5 | 34.1±3.3 | 33±2.1 | 31.6±4.5 | 32±3.7 |  | 34.2±2.8 | 33.8±4.3 | 31.8±3.3 | 31.4±5.3 |  |
| **INL** | *n-* | 36.7±1.9 | 40.1±2.7 | 38.5±1.2 | 37.1±2.3 | 37.3±3.3 | * | 39.1±2.5 | 41.7±2.6 | 37.3±2.7 | 37±2.3 | * |
|  | *i-* | 36.8±2 | 37.6±3.3 | 38±2.2 | 36.4±2.9 | 36.7±1.4 |  | 36.8±3.8 | 38.8±1.8 | 36.5±3 | 36.4±3 |  |
|  | *t-* | 36.1±2.3 | 37.3±2.6 | 36.7±2 | 34.9±2.3 | 35.3±2 |  | 37±2.9 | 37.7±2.3 | 35.2±2.3 | 34.7±2.4 |  |
|  | *s-* | 36.2±1.8 | 37.8±3.8 | 37±1.6 | 34.8±2.8 | 35.5±2.5 | * | 37.6±4.1 | 38±3.7 | 35.2±3 | 34.6±2.8 |  |
| **OPL** | *n-* | 29.6±4.4 | 33.3±6.9 | 28.8±3.6 | 33.4±4.2 | 30.3±6 |  | 32.7±3.4 | 34.3±10.9 | 36.2±4.9 | 31.8±2.8 |  |
|  | *i-* | 30.4±3.8 | 29.6±3 | 29±2.3 | 31.4±3.6 | 29.3±3.1 |  | 29.4±3.3 | 30±2.7 | 32±5.1 | 31.1±2.6 |  |
|  | *t-* | 26.8±1.8 | 27.4±1.9 | 28.3±3 | 27.8±3 | 28±2 |  | 26.3±1.3 | 29.3±1.2 | 27.8±2.3 | 27.8±3.4 |  |
|  | *s-* | 27.1±2.4 | 28.3±3.5 | 27.4±4.1 | 27.1±1.7 | 28.2±3.3 |  | 27.7±2.3 | 29.2±5.1 | 26.8±1.3 | 27.2±1.9 |  |
| **ONL** | *n-* | 56.1±2.9 | 61.7±13.9 | 60.4±9 | 58.9±9.4 | 59±11.3 |  | 57.4±15.1 | 68.8±8.7 | 53.8±12.5 | 62±5.7 | * |
|  | *i-* | 50.9±5.5 | 61.3±9.6 | 56.9±5.2 | 56.5±9.6 | 55.8±10.7 | * | 58±10.9 | 66.8±2.2 | 54.8±11 | 57.5±9.2 | * |
|  | *t-* | 56.4±5.5 | 65.2±6.1 | 56.9±5.3 | 61.4±5.8 | 57.3±4.5 | * | 63.3±6.8 | 68.3±3 | 61.2±6.2 | 61.6±5.9 | * |
|  | *s-* | 62.6±5.1 | 70.8±8.6 | 63.1±5.4 | 67.7±5.1 | 63.5±4.7 | * | 68.4±9.9 | 74.8±4.2 | 66.8±6.7 | 68.2±4.1 | * |
| **RPE** | *n-* | 12.7±1.1 | 12.5±1.6 | 13.1±1.7 | 13.1±1 | 13.3±1.9 |  | 12.1±1.4 | 13.2±1.8 | 13±1.4 | 13.2±0.8 |  |
|  | *i-* | 11.6±1 | 12.4±1.2 | 12.4±1.3 | 12.2±0.9 | 12.5±1.2 |  | 12±1.2 | 13±0.9 | 12.3±1.2 | 12.1±0.7 |  |
|  | *t-* | 11.1±1 | 11.9±1.9 | 11.2±1.3 | 11.3±0.9 | 11.5±0.8 |  | 11.6±2.2 | 12.5±1.5 | 11±1.3 | 11.5±0.7 |  |
|  | *s-* | 11.9±0.8 | 12.8±1.5 | 12.6±1.3 | 12.4±1.2 | 12.5±1 |  | 12.3±1.2 | 13.5±1.9 | 12.8±1.5 | 12.2±0.9 |  |
| CC – congenital cataract; DC – developmental cataract; C – controls; CCc – CC contralateral eyes; DCc – DC contralateral eyes; bCC – bilateral CC; uCC – unilateral CC; bDC – bilateral DC; uDC – unilateral DC; TOT – total retinal thickness; NFL – nerve fibers layer; GCL – ganglion cell layer; IPL – inner plexiform layer; INL – inner nuclear layer; OPL – outer plexiform layer; ONL – outer nuclear layer; RPE – Retinal Pigment epithelium; n- nasal; i- inferior; t- temporal; s- superior; *statistically significant. | | | | | | | | | | | | |
