# Supplementary material for: Long-term effects of early/late-onset visual deprivation on macular and retinal nerve fibers layer structure: A pilot study
Source: PLoS One. 2023 Mar 23;18(3):e0283423. doi: 10.1371/journal.pone.0283423 (PMC10035877; doi:10.1371/journal.pone.0283423)
Supplement: S2 File — (DOCX) [file pone.0283423.s002.docx]

| **S2 –** Complete RNFL thickness mean values at 3.5, 4.1 and 4.7mm. | | | | | | | | | | | |
| --- | --- | --- | --- | --- | --- | --- | --- | --- | --- | --- | --- |
|  | **C** | **CC** | **CCc** | **DC** | **DCc** |  | **uCC** | **bCC** | **uDC** | **bDC** |  |
| **3.5mm** | | | | | | | | | | | |
| g- | 98.9±7.9 | 104.2±12.1 | 109.9±10.4 | 99±15.4 | 97.8±18.6 |  | 108.3±7 | 100.1±15.1 | 93.8±13.8 | 101.9±16.2 |  |
| n- | 79.9±9.2 | 86.9±17.6 | 91±10.9 | 81.9±17.2 | 86±19.2 |  | 96±13.2 | 77.7±17.5 | 84±18.7 | 80.8±17.3 |  |
| ni- | 113.4±20.1 | 114.1±21.3 | 119.6±17.4 | 115.6±34.8 | 133.2±36.7 |  | 114±23.5 | 114.1±20.8 | 112.2±51.3 | 117.4±25.2 |  |
| ti- | 142.4±38.7 | 157.7±19.2 | 159.3±10.6 | 148.8±28.5 | 135.2±23.9 |  | 153.4±13.4 | 162±24.1 | 128.4±13.6 | 160.1±28.7 |  |
| t- | 70.7±8.2 | 71.2±12.8 | 69.6±10.8 | 68.3±15.7 | 63±10.2 |  | 75.1±7.8 | 67.3±16.2 | 64.4±11 | 70.4±18.1 |  |
| st- | 131.7±23.3 | 131.2±22.2 | 131.9±17.9 | 127±26.5 | 110.2±14.7 |  | 137.1±16.5 | 125.3±26.7 | 105.6±17.5 | 138.9±23.2 |  |
| sn- | 110.9±14 | 137.7±22.7 | 137.6±22.1 | 119.8±20.1 | 125.6±30.5 | * | 139.4±27.6 | 136±18.5 | 121.4±21.7 | 118.9±20.4 | * |
|  | | | | | | | | | | | |
| **4.1mm** | | | | | | | | | | | |
| g- | 83.6±6.9 | 88.7±9.8 | 90±5.2 | 84.1±12 | 82.4±15.6 |  | 92.4±4 | 85±12.6 | 80.2±10.8 | 86.3±12.6 |  |
| n- | 66±7 | 72.5±14.4 | 73.6±10.3 | 67.1±12.2 | 67.6±14.9 |  | 80±10.3 | 65±14.5 | 67±13.1 | 67.2±12.5 |  |
| ni- | 89.4±16.7 | 92.4±12.4 | 92.3±13.7 | 91.1±26.9 | 105.4±28.2 |  | 93.9±11.2 | 91±14.2 | 92.8±40.3 | 90.1±19.1 |  |
| ti- | 137.5±16.1 | 138.9±18.6 | 145.6±12.3 | 132.6±24 | 122.6±22.9 |  | 137.6±13 | 140.1±24.1 | 119.8±15.8 | 139.8±25.5 |  |
| t- | 61.9±6.5 | 63.3±13.1 | 60.3±9.3 | 61±14.6 | 58.8±10.4 |  | 66.4±12 | 60.1±14.4 | 58±11.4 | 62.7±16.6 |  |
| st- | 117.5±17.6 | 119.7±22.6 | 122.9±17.8 | 114.6±23.3 | 97±20.1 |  | 124.9±19.7 | 114.6±25.6 | 95.8±15.7 | 125.1±20.3 |  |
| sn- | 88.1±13 | 107.6±19.5 | 111.7±17.5 | 97.9±16.4 | 101±27.7 | * | 109.1±24.2 | 106.1±15.4 | 101±21.2 | 96.2±14.2 |  |
|  | | | | | | | | | | | |
| **4.7mm** | | | | | | | | | | | |
| g- | 72.6±5.9 | 79±9.5 | 78.9±4.1 | 74.4±10.2 | 72.4±12.7 |  | 83.9±5.1 | 74.1±10.8 | 71.4±9.1 | 76±10.9 |  |
| n- | 56.2±6.4 | 63.1±12.9 | 63.9±7.9 | 58.3±10.4 | 58.6±14.3 |  | 70.6±8.8 | 55.6±12.3 | 59±10.2 | 57.9±11.1 | * |
| ni- | 71.5±12.6 | 77.6±10.7 | 74.9±9.1 | 73.1±20.5 | 89.6±23.7 |  | 79.7±7.7 | 75.4±13.4 | 73.8±30.9 | 72.7±14.4 |  |
| ti- | 120.6±12.9 | 125.6±16 | 129.7±15.8 | 119.6±21.7 | 109.4±21.5 |  | 125.4±10.5 | 125.9±21.1 | 107.6±16.8 | 126.3±21.9 |  |
| t- | 57.1±5.3 | 58.9±13.1 | 54.6±8.9 | 56.6±12.8 | 53±8.8 |  | 62.7±13.3 | 55.1±12.8 | 54.6±9.8 | 57.8±14.6 |  |
| st- | 108.1±13.7 | 110±18.6 | 113±15.4 | 108.3±21.7 | 89.6±17.9 |  | 115.3±15.5 | 104.7±21 | 91.8±17 | 117.4±18.8 |  |
| sn- | 70.8±10.1 | 93.4±19.2 | 92.1±13.9 | 81.7±13.6 | 83.6±21.9 | * | 101±21.9 | 85.7±13.3 | 85.8±16.4 | 79.4±12.2 | * |
| CC – congenital cataract; DC – developmental cataract; C – controls; CCc – CC contralateral eyes; DCc – DC contralateral eyes; bCC – bilateral CC; uCC – unilateral CC; bDC – bilateral DC; uDC – unilateral DC; g- global; n- nasal; ni- nasal inferior; ti- temporal iniferior; t- temporal; st- superotemporal; sn- superonasal; *statistically significant. | | | | | | | | | | | |
