# Supplementary material for: Long-term effects of early/late-onset visual deprivation on macular and retinal nerve fibers layer structure: A pilot study
Source: PLoS One. 2023 Mar 23;18(3):e0283423. doi: 10.1371/journal.pone.0283423 (PMC10035877; doi:10.1371/journal.pone.0283423)
Supplement: S3 File — (DOCX) [file pone.0283423.s003.docx]

**S3** – Significant correlations between Retinal layers thickness, RNFL thickness, BCVA and age at surgery.

|  |  |  | **CC** | | | | | | |  |  | **DC** | | | | | | | | |
| --- | --- | --- | --- | --- | --- | --- | --- | --- | --- | --- | --- | --- | --- | --- | --- | --- | --- | --- | --- | --- |
|  |  |  | 3mm | | | | 6mm | | |  |  | 1mm | 3mm | | | | 6mm | | | |
|  |  |  | *n-* | *i-* | *t-* | *s-* | *n-* | *i-* | *t-* |  |  |  | *n-* | *i-* | *t-* | *s-* | *n-* | *i-* | *t-* | *s-* |
| **Rt/BCVA** | **TOT** | R | -0,5726 | -0,6281 |  |  | -0,5664 |  |  | **TOT** | R | 0,5780 |  |  |  |  |  |  |  |  |
|  |  | P | 0,0204 | 0,0092 |  |  | 0,0222 |  |  |  | P | 0,0190 |  |  |  |  |  |  |  |  |
|  | **NFL** | R |  |  | 0,6021 |  |  |  | 0,6635 | **GCL** | R | 0,6044 |  |  |  |  |  |  |  |  |
|  |  | P |  |  | 0,0136 |  |  |  | 0,0050 |  | P | 0,0131 |  |  |  |  |  |  |  |  |
|  | **GCL** | R |  |  |  |  |  |  | -0,7050 | **IPL** | R | 0,5891 |  |  |  |  |  |  |  |  |
|  |  | P |  |  |  |  |  |  | 0,0023 |  | P | 0,0163 |  |  |  |  |  |  |  |  |
|  | **IPL** | R |  |  |  |  | -0,5257 |  | -0,5499 | **OPL** | R | 0,6026 |  |  |  |  |  |  |  |  |
|  |  | P |  |  |  |  | 0,0365 |  | 0,0273 |  | P | 0,0135 |  |  |  |  |  |  |  |  |
|  | **OPL** | R |  |  |  |  |  |  | -0,5424 |  |  |  |  |  |  |  |  |  |  |  |
|  |  | P |  |  |  |  |  |  | 0,0299 |  |  |  |  |  |  |  |  |  |  |  |
|  | **RPE** | R | -0,5365 | -0,5227 | -0,5201 | -0,5213 |  |  |  |  |  |  |  |  |  |  |  |  |  |  |
|  |  | P | 0,0321 | 0,0378 | 0,0389 | 0,0384 |  |  |  |  |  |  |  |  |  |  |  |  |  |  |
| **Rt/AS** | **TOT** | R | -0,5288 | -0,6747 |  |  |  |  |  | **TOT** | R |  |  |  |  |  | -0,6478 | -0,5406 |  | -0,5999 |
|  |  | P | 0,0359 | 0,0041 |  |  |  |  |  |  | P |  |  |  |  |  | 0,0066 | 0,0306 |  | 0,0140 |
|  | **NFL** | R |  |  | 0,7701 |  |  |  | 0,7533 | **NFL** | R |  |  |  |  |  |  | -0,5545 | -0,5776 | -0,5662 |
|  |  | P |  |  | 0,0005 |  |  |  | 0,0007 |  | P |  |  |  |  |  |  | 0,0258 | 0,0191 | 0,0222 |
|  | **GCL** | R |  |  |  |  | -0,5095 |  | -0,6317 | **GCL** | R |  | -0,5885 | -0,5995 | -0,5268 | -0,6109 | -0,7742 | -0,5837 | -0,5747 | -0,6749 |
|  |  | P |  |  |  |  | 0,0438 |  | 0,0087 |  | P |  | 0,0210 | 0,0141 | 0,0360 | 0,0119 | 0,0004 | 0,0176 | 0,0199 | 0,0041 |
|  | **IPL** | R |  |  |  |  | -0,5777 |  |  | **IPL** | R |  | -0,5978 | -0,5640 |  | -0,6369 | -0,7735 | -0,6113 | -0,7244 | -0,7117 |
|  |  | P |  |  |  |  | 0,0191 |  |  |  | P |  | 0,0186 | 0,0228 |  | 0,0080 | 0,0004 | 0,0119 | 0,0015 | 0,0020 |
|  | **OPL** | R |  |  | -0,5343 |  |  |  | -0,5569 | **INL** | R | 0,5581 | 0,7851 | 0,5407 |  | 0,6147 |  |  |  |  |
|  |  | P |  |  | 0,0330 |  |  |  | 0,0250 |  | P | 0,0246 | 0,0005 | 0,0306 |  | 0,0113 |  |  |  |  |
|  | **RPE** | R |  |  |  | -0,5145 |  |  |  | **RPE** | R | -0,5982 |  |  |  |  |  |  |  |  |
|  |  | P |  |  |  | 0,0414 |  |  |  |  | P | 0,0144 |  |  |  |  |  |  |  |  |
|  |  |  | **3.5mm** | | **4.1mm** | | | **4.7mm** | |  |  | **3.5mm** | | | **4.1mm** | | | **4.7mm** | | |
| **RNFLt/AS** | ***n-*** | R |  |  |  | 0,5542 |  |  |  | ***g-*** | R |  | -0,6434 |  |  |  |  |  | -0,6450 |  |
|  |  | P |  |  |  | 0,0397 |  |  |  |  | P |  | 0,0130 |  |  |  |  |  | 0,0127 |  |
|  |  |  |  |  |  |  |  |  |  | ***n-*** | R |  |  |  |  |  |  |  | -0,5614 |  |
|  |  |  |  |  |  |  |  |  |  |  | P |  |  |  |  |  |  |  | 0,0367 |  |
|  |  |  |  |  |  |  |  |  |  | ***ti-*** | R |  | -0,5273 |  |  | -0,5560 |  |  |  |  |
|  |  |  |  |  |  |  |  |  |  |  | P |  | 0,0526 |  |  | 0,0373 |  |  |  |  |
| **RNFLt/BCVA** | ***sn-*** |  |  |  |  |  |  |  | 0,8523 |  |  |  |  |  |  |  |  |  |  |  |
|  |  |  |  |  |  |  |  |  | 0,0148 |  |  |  |  |  |  |  |  |  |  |  |

**R –** correlation coefficient; **P –** p-value; **Rt –** Retinal thickness; **BCVA –** best corrected visual acuity; **AS –** age at surgery; **RNFLt –** retinal nerve fibers layer thickness; **CC –** congenital cataract; **DC –** developmental cataract; ***n-*** nasal; ***i-*** inferior; ***t-*** temporal; ***s-*** superior; ***g-*** global; ***sn-*** superonasal; ***ti-*** temporal inferior.
